# Dysfunctional Cognition in Individuals With an Increased Risk for Mania

**DOI:** 10.32872/cpe.3733

**Published:** 2021-03-10

**Authors:** Raphaela Ulrich, Thomas D. Meyer, Sylke Andreas, Claudia Lex

**Affiliations:** a Department of Psychology, University of Klagenfurt, Klagenfurt, Austria; b Faillace Department of Psychiatry and Behavioral Sciences, McGovern School of Medicine, University of Texas HSC, Houston, TX, USA; c Department of Psychology, University Witten/Herdecke, Witten, Germany; d Department of Psychiatry, Villach General Hospital, Villach, Austria; University of Hamburg, Hamburg, Germany

**Keywords:** bipolar disorder, hypomania, hypomanic personality, dysfunctional attitudes, cognition, vulnerability

## Abstract

**Background:**

There is still a lack of knowledge about attitudes and cognitions that are related to bipolar disorder. Theoretically, it was proposed that exaggerated beliefs about the self, relationships, the need for excitement, and goal-related activities might lead to mania in vulnerable individuals, however, the few studies that examined this hypothesis provided mixed results. One of the unresolved issues is if such a cognitive style is associated with current mood symptoms or with different stages of the illness, i.e. at-risk versus diagnosed bipolar disorder. Therefore, the present study aimed at evaluating depression and mania-related cognitive style in individuals at-risk for mania.

**Method:**

In an online survey, we collected data of 255 students of the University of Klagenfurt, Austria. All participants completed the Hypomanic Personality Scale (HPS), the Cognition Checklist for Mania – Revised (CCL-M-R), the Dysfunctional Attitude Scale (DAS), the Beck Depression Inventory (BDI), and the Internal State Scale (ISS).

**Results:**

In a hierarchical regression, HPS was positively related to scores of all subscales of the CCL-M-R. The HPS did not significantly predict scores of the DAS. Current manic and depressive symptoms significantly contributed to the models.

**Conclusion:**

The present results suggest that a trait-like risk for mania is associated with mania-related but not depression-related cognitions.

## Abstract (highlights)

- Individuals at-risk for mania show mania-specific rather than depression-specific thinking patters.
- Current subclinical mood symptoms are related to mood-congruent attitudes and cognitions.

Bipolar spectrum disorders, which include bipolar I, bipolar II, and subthreshold bipolar disorder, affect about 2.4% of the population worldwide (Merikangas et al., 2011) and can be highly disabling. Compared to other psychiatric illnesses, bipolar disorder (BD) is the fifth leading cause of years lived with disability (Ferrari et al., 2016), it is associated with social disruption (e.g., Depp et al., 2010) and an increased risk of suicide (e.g., Nordentoft et al., 2011). Psychological treatments for BD combined with pharmacological strategies yielded better outcomes than pharmacological treatment alone (Miklowitz et al., 2007). For example, structured psychological treatments, such as cognitive behavioral therapy (CBT) seem to be effective (Chiang et al., 2017), but this effect might be specific for depressive symptoms (Oud et al., 2016). One reason for this result might be that cognitive behavioral interventions for BD stem from CBT that was originally developed in the context of major depression (Lam et al., 2010), and usually psychotherapy does not focus on decreasing activation, changing self-confident thoughts or lowering elevated mood. In addition, there is still relatively little knowledge about cognition specifically related to BD and mania.

One of the few cognitive theories specifically developed for BD was proposed by Beck et al. (2006). They state that individuals possess schemata defined as underlying cognitive structures for organizing perceptions of the world. These schemata can be detected by asking people about their beliefs and attitudes. If a negatively biased schema is activated by a stressful life event, the individual might develop even more negative thoughts and subsequently experience depressive symptoms. For example, an underlying belief “I am incompetent” can be represented in the conscious thought “I can’t do it” when asked to handle a difficult situation, which then might lead to an increase in depressive symptoms (Beck & Haigh, 2014). Parallel, a different set of dysfunctional beliefs might lead to manic episodes. These mania-specific cognitions relate to exaggerated beliefs about self-worth, to grandiose beliefs about interpersonal relationships, to erroneous beliefs about needing excitement caused by high-risk situations, and unrealistic beliefs about having high energy levels for undertaking goal-driven activities (Beck et al., 2006; Newman et al., 2002).

To tap into mania-related dysfunctional beliefs Beck et al. (2006) developed the Cognition Checklist for Mania – Revised (CCL-M-R) that comprises four subscales, i.e. ‘Myself’, ‘Relationship’, ‘Pleasure/Excitement’, and ‘Activity’. Based on Beck’s model, all four dimensions of the CCL-M-R should be elevated in manic states. However, the few studies conducted so far have yielded mixed results. Beck et al. (2006) found that currently manic patients indeed reported more mania-related cognition with regards to ‘Myself’, ‘Relationship’, and ‘Activity’ compared to patients in depressed and mixed states, whereas another study found that only the ‘Pleasure/Excitement’ subscore was related to manic symptoms (Fulford et al., 2009). In addition, it is unclear whether this specific set of cognitions is associated exclusively with manic states or if they persist in other bipolar states as well, e.g., remission or prodromal. While two studies mentioned before concluded that certain mania-related cognitions were linked only to acute manic states (Beck et al., 2006; Fulford et al., 2009; Ruggero et al., 2015) showed that individuals with a history or current diagnosis of BD reported elevated levels of mania-related cognitions, irrespective of current symptoms. Also, mania-related cognitions might be present and prevalent in different stages of the disorder, i.e. at-risk stages or symptomatic BD (Fulford et al., 2009). For example, beliefs relating to self-confidence in the CCL-M-R were increased in individuals at risk for BD but not in those diagnosed with BD. In contrast, cognitions relating to interpersonal problems were increased in individuals diagnosed with BD but not in those at high risk for BD.

A few more studies examined depression-related cognition in BD. In this context, one of the most wildly used instruments is the Dysfunctional Attitude Scale (DAS; Weisman, 1979). However, the results of the studies that used the DAS were mixed. Some studies found no differences in overall dysfunctional attitudes between healthy controls and individuals diagnosed with remitted BD (Alatiq et al., 2010; Lex et al., 2008; Lex et al., 2011; Mansell et al., 2011). Other studies found elevated DAS scores in patients with remitted BD relative to healthy control groups (Hollon et al., 1986; Jones et al., 2005; Scott et al., 2000; Tosun et al., 2015). However, dysfunctional attitudes refer to different areas, for example, achievement, dependency, and goal attainment. Since mania involves increased goal-directed activity (American Psychiatric Association, 2013) some researchers argued that it would be essential to focus on assessing beliefs relating to goal attainment. In line with this, Lam et al. (2004) found evidence that dysfunctional attitudes related to goal attainment were indeed more pronounced in individuals with BD compared to unipolar depression. However, this was not found in all studies (e.g., Jabben et al., 2012).

Despite the recent increased efforts to understand cognitive processes in BD, studies are still sparse and their results are mixed. For example, it still remains unclear whether these dysfunctional cognitions are tied to depressive or (hypo)manic states of BD or if they are part of the underlying diathesis of BD. One possibility to examine this question would be to assess these cognitions among individuals at-risk for mania. Risk for BD can be defined via a genetic vulnerability (Ruggero et al., 2015) or via a constitutional predisposition. Hyperthymic temperament represents such a constitutional predisposition for mania and can be assessed by the Hypomanic Personality Scale (HPS; Eckblad & Chapman, 1986) because there is evidence that people scoring high on the HPS are more likely to develop symptoms of BD over time (Blechert & Meyer, 2005; Kwapil et al., 2000; Walsh et al., 2015).

Therefore, the present study aimed at examining, if mania related cognition depicted by the CCL-M-R were even present in at-risk states or if they were rather tied to acute manic symptoms. Also, we were interested if core beliefs related to goal attainment were associated with at-risk states for mania. Therefore, we hypothesized that risk for mania predicted mania-related cognition assessed with the CCL-M-R and the DAS subscale ‘Goal Attainment’. We also expected that current manic symptoms were associated with mania-related cognition. We, however, did not expect such a relation for depression-specific cognition, i.e. DAS-subscales ‘Dependency’ and ‘Achievement’.

## Method

### Participants and Procedure

At first, we contacted all students at the University of Klagenfurt, Austria, via their campus e-mail addresses. The e-mail contained general information on the study and a link to “Lime Survey”. “Lime Survey” is a web application to conduct online surveys. If the students decided to participate, the provided informed consent, filled out the questionnaires, and provided demographic data. We also asked if they had been in psychotherapy before, because some psychological approaches might potentially alter cognitions related to mood symptoms. The participants remained anonymous and could leave the survey and delete their data at any time. At the end, the participants could optionally disclose their mail address to obtain course credit (*n* = 68). In total, we obtained data from 255 students. Most participants were female (80%) and had never been in psychotherapy before (63.5%). The demographic data is displayed in Table 1.

**Table 1:** Characteristics of the Sample (N = 255)

Variable	*M*	*SD*	*Minimum*	*Maximum*
Age	28.27	9.56	18	65
HPS	16.28	8.25	1	42
BDI	10.25	9.36	0	48
ACT	142.88	95.22	0	466
CCL-M-R				
Myself	8.42	4.12	1	19
Relation	4.05	2.92	0	14
Pleasure/ Excitement	9.87	4.00	0	20
Activity	7.80	3.39	0	18
Thwarting	0.90	1.38	0	6
Total	30.14	11.12	1	60
DAS				
Goal Attainment	20.27	4.82	3	35
Dependency	8.27	4.84	0	22
Achievement	9.80	6.60	0	29
Total	60.58	18.03	20	117

*Note.* ACT = Internal State Scale Activation Subscore; BDI = Beck Depression Inventory; CCL-M-R = Cognition Checklist Mania; DAS-24 = Dysfunctional Attitude Scale; HPS = Hypomanic Personality Scale.

### Measurements

#### Hypomanic Personality Scale (HPS)

The HPS (Eckblad & Chapman, 1986) is a self-rating scale and includes 48 true-false items covering emotions (e.g., “I frequently get into moods where I feel very speeded-up and irritable”), behavior (e.g., “At social gatherings, I am usually the ‘life of the party’”), and energy level, (e.g., “There have often been times when I had such an excess of energy that I felt little need to sleep at night”) one feels at most times of his/her life. It assesses hyperthymic temperament, was used in clinical and non-clinical samples before, and is predictive of bipolar disorder and (hypo)manic symptoms (Blechert & Meyer, 2005; Kwapil et al., 2000; Walsh et al., 2015). In the present study, we used the total score to operationalize a constitutional risk to develop mania. Scores can range between 0 and 48, and individuals scoring above 26 are considered at high risk for mania (Meyer & Baur, 2009). The German version (Meyer et al., 2000) showed an internal consistency of α = .89. Hyperthymic temperament was a stable trait over time (*r*_tt_ = .87 [2 years]; Hofmann & Meyer, 2006). In the present sample the reliability was adequate (Cronbach’s α = .87), and 34 participants were considered at high risk for mania (HPS > 26).

#### Cognition Checklist for Mania – Revised (CCL-M-R)

The CCL-M-R (Beck et al., 2006; Goldberg et al., 2008) includes 29-items assessing beliefs associated with mania one had had during the past two days and has been used in clinical and non-clinical samples (Fulford et al., 2009). The questionnaire contains four subscales. The ‘Myself’ subscale contains 7 items and assesses cognition related to the self (e.g., “I am the best”), the ‘Relationship’ subscale contains 7 items and assesses interpersonal issues (e.g., “I love everyone”), the ‘Pleasure/Excitement’ scale contains 9 items exploring excitement seeking (e.g., “It is OK to take risks”), and the ‘Activity’ subscale comprises 6 items and assesses goal-driven activities (e.g., “I have got to get the job done while I can”). A ‘Thwarting’ subscale can be derived from the ‘Relationship’ scale by summing two items (“I could accomplish great things, if people did not get in my way” and “Other people stand between me and my goals”; Fulford et al., 2009). Independent back-translation was used by two of the authors (R. U. and C. L.) and a native English speaker to obtain a German version of the CCL-M-R. Internal consistencies in the present study were adequate (Total CCL-M-R score: Cronbach’s α = .89, ‘Myself’: α = .80, ‘Relationship’: α = .64, ‘Pleasure/Excitement’: α = .83, ‘Activity’: α = .70) and comparable to the English version (Beck et al., 2006; Ruggero et al., 2015).

#### Dysfunctional Attitude Scale (DAS-24)

The DAS (Weisman, 1979; German version: Hautzinger et al., 1985) is designed to assess depression-specific beliefs that individuals have about themselves, others, and their environments most of the time. Although the DAS has been widely applied in clinical samples, it is also used with analogue samples (e.g., Perez & Rohan, 2021). Lam et al. (2004) used a 24-items DAS version in their study from which 3 factors could be derived: ‘Goal Attainment’ (6 items e.g., “I ought to be able to solve problems quickly”), ‘Dependency’ (4 items, e.g., “If others dislike you, you cannot be happy”, and ‘Achievement’ (5 items, e.g., “People who have good ideas are more worthy”). These subscales showed good internal consistency. In the present study, we adapted the German DAS in order to parallel the DAS-24 by Lam et al. (2004). We obtained Cronbach α = .83 for the total score, for ‘Goal Attainment’ α = .44, for ‘Dependency’ α = .65, and for ‘Achievement’ α = .80.

#### Beck Depression Inventory (BDI)

The BDI (Beck et al., 1961; German version: Hautzinger et al., 1995) measures the severity of self-reported depression during the past 2 weeks and is used in clinical and non-clinical samples (Richter et al., 1998). It consists of 21 items and each item is scored on a 4-point scale, e.g. “0 - I do not feel sad; 1 - I feel sad; 2 - I am sad all the time and I can't snap out of it, 3 - I am so sad and unhappy that I can't stand it”. Scores can range from 0 to 63, and higher scores indicate more severe depressive symptoms. In the present study, we used the validated German version that has comparable psychometric properties to the English version (Hautzinger et al., 1995).

#### Internal State Scale (ISS)

The ISS (Bauer et al., 1991, 2000; German version: Meyer & Hautzinger, 2004) is a self-report measure that consists of 16 items that are rated on a visual analogue scale (0 – “not at all” to 100 – “totally”) incorporating 4 subscales (Activation, Well Being, Perceived Conflict, Depression Index). The Activation subscale (ACT) contains 5 items. It reflects self-reports of manic symptoms within the last 24 hours by assessing behavioral and formal cognitive activation (e.g. “I feel overactive”, “My thoughts are going fast”). It correlates positively with self- and expert ratings of mania (Bauer et al., 1991, 2000) and has been used in clinical and non-clinical samples (e.g., Kelly et al., 2016).

#### Statistical Analysis

To examine if high risk for mania predicted depression- and mania-specific cognition we calculated hierarchical regression analyses using IBM SPSS Statistics for Macintosh, Version 25.0. Scores of the CCL-R-M and DAS-24 were used as dependent variables. All analyses controlled for age and gender in Block 1, for current manic and depressive symptoms in Block 2, and for a prior history of psychotherapy in Block 3. Scores of the HPS were entered in Block 4 after accounting for the other variables of interest. Prior to interpreting the models, the relevant assumptions for linear regressions and potential biases were examined. First, the visual inspection of all scatter plots depicturing ‘standard residuals’ vs. ‘standard predicted value’ revealed no specific pattern, hence the assumptions of linearity and heteroscedasticity were met. Second, the correlations between the predictors were low (all *r* < |.5|), and the multicollinearity statistics (i.e., Tolerance and VIF) were all within the tolerable limits (Field, 2009). Third, histograms and P-P plots showed that the standard residuals were normally distributed. Forth, the assumption of independent errors was met because all Durbin-Watson results were close to 2 (between 1.83 and 2.14). Finally, we identified the presence and significance of outliers by looking at the standard residuals, the Mahalanobis distance and the leverage effect (i.e., Cook’s distance). Cases with standard residuals values below -2 and above 2 were defined as outliers. However, the proportion of identified outliers was less than 5% in all analyses and was, therefore, tolerated (Field, 2009). In order to examine this issue in more detail, we also looked at the Mahalanobis distance. Eleven cases were defined as outliers because their values of the Mahalanobis distance were above 22.59 (for the cut-off value see Stevens, 1984). However, the leverage effects of these 11 cases were small (i.e., Cook’s distance < 1); therefore, the cases were not deleted from the analyses (Field, 2009, p. 309).

## Results

First, the final overall models including all predictors for *mania-related cognitions* (CCL-M-R) are reported. The final overall model for the composite CCL-M-R score was significant *F*(6, 248) = 23.96, *p* < .001. Also, the final overall models for the specific dimensions of the CCL-M-R were significant: ‘Myself’ *F*(6, 248) = 22.56, *p* < .001, ‘Relationship’ *F*(6, 248) = 18.05, *p* < .001, ‘Pleasure/Excitement’ *F*(6, 248) = 14.52, *p* < .001, and ‘Activity’ *F*(6, 248) = 15.40, *p* < .001. Looking at the Δ*R*^2^, it became evident that BDI, ACT, and HPS scores significantly increased the explained variance in all five models (Table 2).

**Table 2:** Final Model (Step 4) of the Hierarchical Regression Analyses for Cognition Related to Mania

Predictor	CCL-M-R Total			CCL-M-R Myself			CCL-M-R Relationship			CCL-M-R Pleasure/Excitement			CCL-M-R Activity		
	*B*	*SE_B_*	β	*B*	*SE_B_*	β	*B*	*SE_B_*	β	*B*	*SE_B_*	β	*B*	*SE_B_*	β
Block 1															
Sex	2.02	1.45	0.07	0.45	0.54	0.04	0.94	0.40	0.13*	0.06	0.56	0.01	0.56	0.47	0.07
Age	0.06	0.06	0.05	0.00	0.02	0.00	0.02	0.02	0.08	-0.01	0.02	-0.02	0.04	0.02	0.12*
Block 2															
BDI	-0.11	0.06	-0.09	-0.17	0.02	-0.39***	0.12	0.02	0.37***	0.01	0.02	0.01	-0.06	0.02	-0.17**
ACT	0.03	0.01	0.22***	0.01	0.00	0.14**	0.00	0.00	0.05	0.01	0.00	0.20***	0.01	0.00	0.26***
Block 3															
Therapy	0.00	0.00	-0.01	0.00	0.00	0.00	0.00	0.00	-0.05	0.00	0.00	-0.06	0.00	0.00	0.08
Block 4															
HPS	0.65	0.08	0.48***	0.23	0.03	0.46***	0.10	0.02	0.28***	0.18	0.03	0.36***	0.14	0.03	0.35***

*Note.* CCL-M-R Total: *R*^2^ = .01 for Block 1; Δ*R*^2^ = .18*** for Block 2; Δ*R*^2^ = .01 for Block 3; Δ*R*^2^ = .17*** for Block 4; CCL-M-R Myself: *R*^2^ = .01 for Block 1; Δ*R*^2^ = .19*** for Block 2; Δ*R*^2^ = .00 for Block 3; Δ*R*^2^ = .15*** for Block 4; CCL-M-R Relationship: *R*^2^ = .03* for Block 1; Δ*R*^2^ = .21*** for Block 2; Δ*R*^2^ = .01 for Block 3; Δ*R*^2^ = .05*** for Block 4; CCL-M-R Pleasure/Excitement: *R*^2^ = .01 for Block 1; Δ*R*^2^ = .14*** for Block 2; Δ*R*^2^ = .01 for Block 3; Δ*R*^2^ = .10*** for Block 4; CCL-M-R Activity: *R*^2^ = .02 for Block 1; Δ*R*^2^ = .16*** for Block 2; Δ*R*^2^ = .00 for Block 3; Δ*R*^2^ = .09*** for Block 4.

ACT = Internal State Scale Activation Subscore; BDI = Beck Depression Inventory; CCL-M-R = Cognition Checklist Mania; HPS = Hypomanic Personality Scale; Therapy = Prior Psychotherapy.

*p < .05. **p < .01. ***p < .001.

More specifically, ACT positively predicted cognition related to ‘Myself’ (β = 0.14), ‘Pleasure/Excitement’ (β = 0.20), and ‘Activity’ (β = 0.26), BDI positively predicted cognition related to ‘Relationship’ (β = 0.37), and HPS scores positively predicted all CCL-M-R dimensions as well as the total CCL-M-R score. An exploratory hierarchical regression model for the Thwarting subscale was also significant, *F*(6, 248) = 10.63, *p* < .001 (final model). Specifically, BDI (β = 0.40, *p* < .001) and HPS scores (β = 0.16, *p* = .01) predicted Thwarting.

Next, the final overall models including all predictors for *depression-related cognitions* (DAS-24) are reported. The final overall model for the composite DAS-24, *F*(6, 248) = 18.30, *p* < .001, as well as the final overall models for the specific dimensions of the DAS-24 were significant: ‘Achievement’ *F*(6, 248) = 15.20, *p* < .001, ‘Dependency’ *F*(6, 248) = 13.63, *p* < .001, ‘Goal Attainment’ *F*(6, 248) = 4.52, *p* < .001. The BDI significantly predicted attitudes related to ‘Achievement’ (β = 0.45), ‘Dependency’ (β = 0.46) and the total DAS-24 score (β = 0.49). The ACT (β = 0.20) and sex (β = 0.17) significantly predicted ‘Goal Attainment’. The HPS score could not increase the explained variance in any of the regression models (Table 3).

**Table 3:** Final Model (Step 4) of the Hierarchical Regression Analyses for Cognition Related to Depression

Predictor	DAS-24 Total			DAS-24 Achievement			DAS-24 Dependency			DAS-24 Goal Attainment		
	*B*	*SE_B_*	β	*B*	*SE_B_*	β	*B*	*SE_B_*	β	*B*	*SE_B_*	β
Block 1												
Sex	3.98	2.45	0.09	1.66	0.92	0.10	0.13	0.69	0.01	2.06	0.75	0.17**
Age	-0.02	0.10	-0.01	0.02	0.04	0.03	0.00	0.03	-0.01	0.03	0.03	0.06
Block 2												
BDI	0.95	0.11	0.49***	0.32	0.04	0.45***	0.24	0.03	0.46***	0.03	0.03	0.05
ACT	0.02	0.01	0.11	0.01	0.00	0.11	0.00	0.00	0.04	0.01	0.00	0.20**
Block 3												
Therapy	0.00	0.00	0.00	0.00	0.00	-0.03	0.00	0.00	0.04	0.00	0.00	-0.03
Block 4												
HPS	0.13	0.13	0.06	0.06	0.05	0.07	0.05	0.04	0.08	0.04	0.04	0.06

*Note.* DAS-24 Total: *R*^2^ = .02* for Block 1; Δ*R*^2^ = .28*** for Block 2; Δ*R*^2^ = .00 for Block 3; Δ*R*^2^ = .01 for Block 4; DAS-24 Achievement: *R*^2^ = .02 for Block 1; Δ*R*^2^ = .24*** for Block 2; Δ*R*^2^ = .00 for Block 3; Δ*R*^2^ = .01 for Block 4; DAS-24 Dependency: *R*^2^ = .01 for Block 1; Δ*R*^2^ = .23*** for Block 2; Δ*R*^2^ = .00 for Block 3; Δ*R*^2^ = .01 for Block 4; DAS-24 Goal Attainment: *R*^2^ = .03** for Block 1; Δ*R*^2^ = .06*** for Block 2; Δ*R*^2^ = .00 for Block 3; Δ*R*^2^ = .01 for Block 4.

*p < .05. **p < .01. ***p < .001.

## Discussion

The present study examined the relation between an increased risk for mania, current mood symptoms and cognition specifically related to depression and mania. The risk for mania was assessed with the HPS. In line with our hypotheses, risk for mania significantly predicted mania-specific but not depression-specific cognitions. However, while we had expected that risk for mania would be specifically related to one aspect of dysfunctional attitudes, i.e., ‘Goal Attainment’, this was not the case. Current manic and depressive mood also contributed significantly to the regression models.

The association between high risk for mania and elevated levels of mania-specific cognition was proposed by Beck and his colleagues (2006) as a logical extension of the original theory of depression (Beck et al., 1979). In line with this theory, we found that the CCL-M-R total score as well as all subscores were associated with increased vulnerability for mania. Beck et al. (2006) also found evidence for their theory regarding most types of mania-specific cognition, however, they failed to find elevated scores on the CCL-M-R subscale ‘Pleasure/Excitement’. As they point out, they tested inpatients who had few opportunities to engage in exciting, high risk behavior while admitted to the hospital. In contrast, our sample consisted of university students who had much more chances for potentially risky behavior to fulfill their need for excitement. This is consistent with Fulford et al. (2009) who also found that HPS scores were related to a modified ‘Pleasure/Excitement’ score of the CCL-M-R in a college student sample.

The CCL-M-R assesses mania-specific beliefs and although it explicitly asks to focus on the last days, it might capture more long-standing beliefs and attitudes about the self, the interaction with others, the engagement of high risk behavior to feel excitement, and the attainment of high goals. This would explain why an indicator of vulnerability for BD would be related to these beliefs, even after accounting for current symptoms. In contrast, Ruggero et al. (2015) found no difference in CCL-M-R scores between individuals at-risk for mania and those with no elevated risk. There are several differences between the studies. We used continuous scaling, whereas Ruggero et al. (2015) used between group differences, i.e. high-risk group vs. low risk group, which could reduce the variance in the predictor group. In addition, their sample was much smaller and might have lower power.^1^1We thank an anonymous reviewer for these comments. Finally, contrary to Ruggero et al. (2015), we assessed current mood symptoms and found associations to the CCL-M-R, therefore, not differentiating between current symptoms and vulnerability could also affect the results. Finally, it might be that the CCL-M-R and the HPS show some construct overlap. Although designed to tap into emotion, behavior, and energy levels, some items of the HPS might also assess cognition, e.g. “I expect that someday I will succeed in several different professions”.^2^2See Footnote 1.


The same study found that the Hypomanic Attitudes and Positive Prediction Inventory (HAPPI; Mansell, 2006) differentiated between individuals at-risk and those with no elevated risk. The HAPPI assesses hypomania-specific positive and negative appraisals relating to high and low activation internal states, e.g., an emotion one feels in a specific situation (e.g., Kelly et al., 2017). Given the few studies, it remains unclear whether cognition relating to internal states as measured by the HAPPI or cognition potentially relating to more long-standing cognitive factors as measured by the CCL-M-R is more relevant for at-risk stages in BD. Furthermore, the way risk for BD is defined might be essential, as well. In the present study, we focused specifically on risk for mania by assessing temperamental traits (e.g., Blechert & Meyer, 2005; Kwapil et al., 2000), whereas Ruggero et al. (2015) defined the risk for BD genetically (offspring of parents diagnosed with BD). Speculatively, individuals scoring high on the HPS who might never have been exposed to actual BD might be less familiar with its presentation and more likely to endorse items on the CCL-M-R than individuals whose parents have expressed such mania-related attitudes and beliefs while being (hypo)manic. Internal processes, such as appraisals might be less shared with others even if they influence actual behaviors. Or perhaps, offspring of parents with BD might have been exposed to challenging situations due to their parent’s disorder during their childhood and therefore be more cautious to endorse, for example, grandiose statements or behaviors that are considered risky as asked in the CCL-M-R.

In the present study, risk for mania did not predict cognitions related to goal attainment as measured with the DAS. Although Lam et al. (2004) found that the ‘Goal Attainment’ subscale of the DAS differentiated between patients with remitted BD and patients with remitted unipolar depression, most previous studies found little evidence for increased scores on the ‘Goal Attainment’ subscale of the DAS in remitted BD (e.g., Alatiq et al., 2010; Lex et al., 2008). This is interesting because there is evidence that a dysregulation of goal-directed behavior and goal striving is an important aspect in BD (Alloy et al., 2012; Urošević et al., 2008) and life events relating to goal attainment caused increases in manic symptoms (Johnson et al., 2000, 2008; Tharp et al., 2016). Subsequently, it would make sense that individuals at-risk for mania endorse exaggerated believes about goal attainment. In the present study risk for mania predicted elevated scores on the ‘Activity’ subscale of the CCL-M-R but not on the ‘Goal Attainment’ subscale of the DAS. One possible reason for this could be that the items of the DAS ‘Goal Attainment’ subscale are worded more generally, e.g. “I should be happy all the time”, while the items on the CCL-M-R ‘Activity’ scale are targeted at more specific events, e.g., “I have new goals”. Additionally, there is evidence that dysfunctional attitudes might be latent outside of acute mood episodes and must be activated before individuals endorse them (Babakhani & Startup, 2012) or are state-dependent (Alloy et al., 1999; Hollon et al., 1986; Lex et al., 2008, 2011; Reilly-Harrington et al., 1999; Scott et al., 2000). We actually found an association between current manic symptoms and the DAS subscale ‘Goal Attainment’. Although one has to keep in mind that the reliability for the ‘Goal Attainment’ subscale was low, this result suggests that manic mood, rather than risk for mania, might be more closely related to dysfunctional attitudes related to goal attainment.

We also found that current subthreshold manic symptoms predicted the mania-related cognition, even though to a lesser degree than the risk for mania. This is consistent with previous studies (Fulford et al., 2009). However, our data also revealed an unexpected association between current depressed mood and the CCL-M-R subscale ‘Relationship’. This is in conflict to previous evidence and to the theoretical background (Beck et al., 2006; Fulford et al., 2009). It might be that some of the items of the ‘Relationship’ scale might relate to depressed mood, e.g., “People treat me like I am sick” and “They do not understand me”. However, even if only those two items of the ‘Relationship’ scale were extracted, that focus on interpersonal behavior most relevant in BD, namely being thwarted by others in the attainment of goals (Fulford et al., 2009), we still found that the level of depression was a significant predictor.

The present study focused on risk and cognitions associated with mania. However, in most cases BD also includes depressive mood episodes. Based on our results we cannot explain how depressive symptoms might arise, which could be a limitation of the present study. In terms of methodical limitations, first, our data was collected online. This approach bears some disadvantages, e.g., limited control regarding the test setting (Wright, 2005). However, there is evidence that paper-and-pencil and Internet data collection methods are equivalent (Weigold et al., 2013). Second, our participants were not asked if they had been diagnosed with an affective or any other psychiatric illness before or if they were experiencing an acute illness episode at the time of their participation. However, in order to control for psychological problems we asked them if they had ever been in psychological therapy and found no relation to mania-specific cognition. Third, we had a mainly female non-clinical sample that might not be representative of people developing BD. However, several reviews emphasize the relevance of analogous samples to understanding clinical phenomena (Abramowitz et al., 2014; Ehring et al., 2011). At last, we used a hierarchical regression design in a cross-sectional approach because we aimed at examining a directional association. It might be that this approach missed longitudinal developments and changes of our target variables.

Despite these limitations, the present study showed that risk for mania was associated with mania-specific dysfunctional cognition. This finding points toward the importance to identify mania-specific cognitions in early or at-risk states of BD in order to help individuals to question and modify these cognitions to potentially prevent more severe symptoms. Future studies should assess mania-specific beliefs in different phases of BD in order to examine the relation between mania-specific cognitions and current mood, perhaps even looking at specific symptoms, such as elated versus irritable mania. Also, longitudinal studies are highly awaited in order to test if dysfunctional cognitions increase the risk of acute bipolar episodes or if they interact with life events or other factors (e.g., Lex et al., 2017).
